# The Effects of Transcranial Direct Current Stimulation (tDCS) on Multitasking Throughput Capacity

**DOI:** 10.3389/fnhum.2016.00589

**Published:** 2016-11-29

**Authors:** Justin Nelson, Richard A. McKinley, Chandler Phillips, Lindsey McIntire, Chuck Goodyear, Aerial Kreiner, Lanie Monforton

**Affiliations:** ^1^Air Force Research Laboratory, Wright-Patterson Air Force BaseOH, USA; ^2^Department of Biomedical, Industrial, and Human Factors Engineering, Wright State UniversityDayton, OH, USA

**Keywords:** dorsolateral prefrontal cortex (DLPFC), multi-attribute task battery (MATB), transcranial direct current stimulation (tDCS), information throughput capacity, multitasking

## Abstract

**Background:** Multitasking has become an integral attribute associated with military operations within the past several decades. As the amount of information that needs to be processed during these high level multitasking environments exceeds the human operators' capabilities, the information throughput capacity reaches an asymptotic limit. At this point, the human operator can no longer effectively process and respond to the incoming information resulting in a plateau or decline in performance. The objective of the study was to evaluate the efficacy of a non-invasive brain stimulation technique known as transcranial direct current stimulation (tDCS) applied to a scalp location over the left dorsolateral prefrontal cortex (lDLPFC) to improve information processing capabilities during a multitasking environment.

**Methods:** The study consisted of 20 participants from Wright-Patterson Air Force Base (16 male and 4 female) with an average age of 31.1 (SD = 4.5). Participants were randomly assigned into two groups, each consisting of eight males and two females. Group one received 2 mA of anodal tDCS and group two received sham tDCS over the lDLPFC on their testing day.

**Results:** The findings indicate that anodal tDCS significantly improves the participants' information processing capability resulting in improved performance compared to sham tDCS. For example, the multitasking throughput capacity for the sham tDCS group plateaued near 1.0 bits/s at the higher baud input (2.0 bits/s) whereas the anodal tDCS group plateaued near 1.3 bits/s.

**Conclusion:** The findings provided new evidence that tDCS has the ability to augment and enhance multitasking capability in a human operator. Future research should be conducted to determine the longevity of the enhancement of transcranial direct current stimulation on multitasking performance, which has yet to be accomplished.

## Introduction

Human multitasking capabilities are readily becoming a key interest within the research community involving military operations. Within the Air Force, various operations such as remotely piloted and manned aircraft operations require a human operator to monitor and respond to multiple events simultaneously over a long period of time. However, with the monotonous nature of these tasks, the operators' performance may decline shortly after their work shift commences. In a multitasking environment, this decline in performance is a result of information overload (Cheshire, 2015). With an increasing demand to process and respond to critical information, the mental and physical demand endured by the human operator can become overwhelming (Subramanyam et al., 2013). Once the number of events exceeds the operators' cognitive capabilities, the information throughput capacity will reach an asymptote limit. Miller (1956) noted that the throughput capacity asymptote for a single-tasking scenario is referred to as a channel capacity. It is at this theoretical point where an operator can no longer effectively interpret and respond to the incoming information and the overall performance either plateaus or begins to decline. In a previous research study, the results led the investigators to propose that there is a throughput capacity when performing a multi-task known as the multi-attribute task battery or MATB (Camden et al., 2015). However, the data was insufficient to determine its existence. Overcoming such capacity limitations is essential to improving multitasking capabilities.

**Figure 1:**
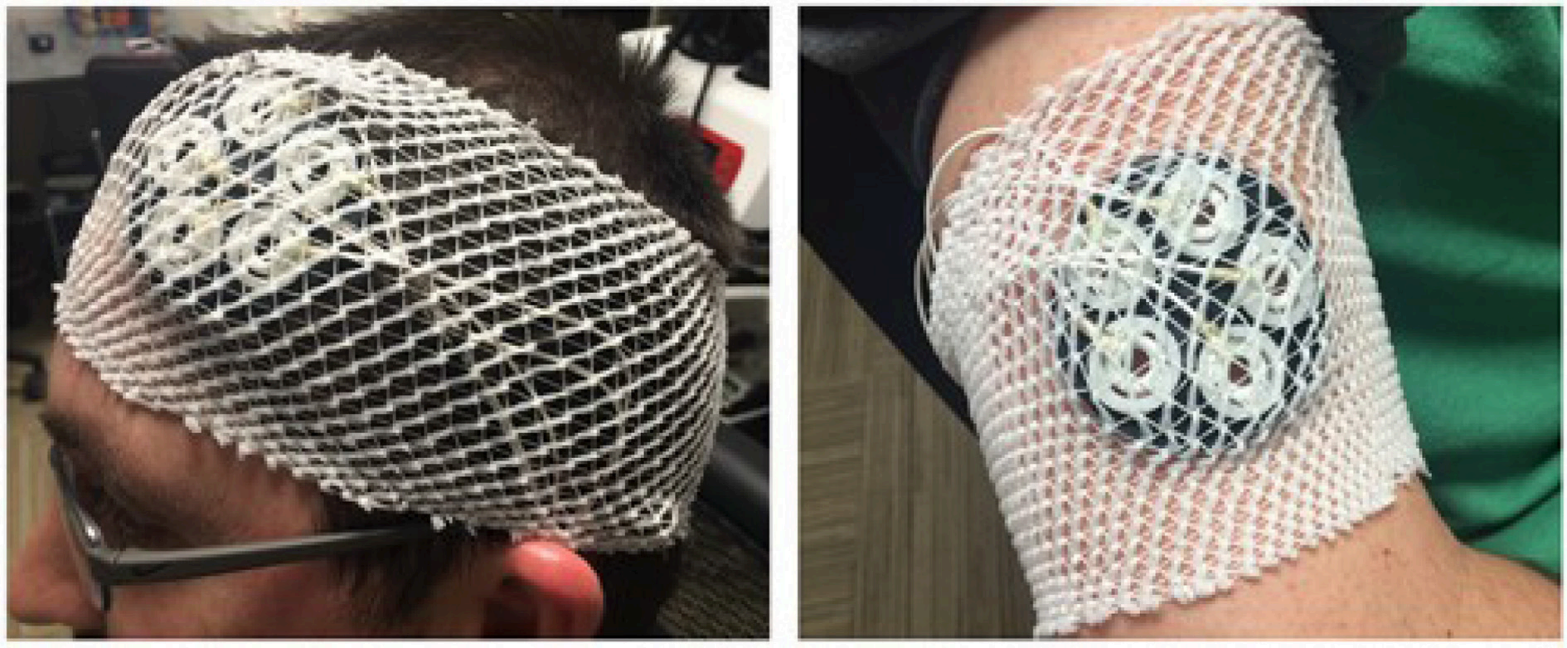
**Electrode placement for the anode (left panel)** and cathode **(right panel)**.

Perhaps one of the most critical aspects of cognition involved with multitasking is attention (Gladwin et al., 2012; Boehm-Davis et al., 2015). Colloquially, stimuli that are not attended to cannot be perceived and processed by the brain. To enhance or direct attention to subtasks within a multitask, countermeasures such as caffeine (Lieberman et al., 2002; Smith, 2002; Harvanko et al., 2015) and haptic feedback have been examined (Camden et al., 2015; Sollfrank et al., 2016) with limited success. However, emerging evidence suggests that a neuromodulatory technique known as transcranial direct current stimulation (tDCS) can enhance aspects of cognition resulting in improved performance during single task operations. For a review of the tDCS technology and technique, see Wagner et al. (2009) or Nitsche et al. (2009). Stimulation can be given prior to, during, or after participants complete a cognitive task. However, a study conducted by Martin found that applying tDCS during a cognitive task provided superior output performance compared to before the task (Martin et al., 2014). A common stimulation site for augmenting cognitive function via tDCS is the dorsolateral prefrontal cortex (DLPFC) which has been associated with working memory, attention, vigilance, planning and reasoning. Anodal tDCS applied over the left or right DLPFC has been previously shown to preserve sustained attention/vigilance performance (i.e., reduce or remove the vigilance decrement), although stimulation over the left also produced a temporary enhancement of performance (McKinley et al., 2012). Follow-on studies evaluated the effects of tDCS over lDLPFC using an electrode montage that placed the cathode over the right shoulder (McIntire et al., 2014; Nelson et al., 2015). Figure 1 provides the specific placements for the anode and cathode. While the cathode is traditionally placed on the scalp, it creates a potential confound due to the fact that cortical excitability is also influenced under this electrode and leads to cognitive effects (Kincses et al., 2004; Penolazzi et al., 2010; Ambrus et al., 2011; Hammer et al., 2011). Hence, moving the cathode off the scalp allows the data to be interpreted in a more straight-forward fashion. The data from McIntire et al. (2014) suggest this extracephalic tDCS montage can initially enhance and then preserve vigilance performance for approximately 6 h (McIntire et al., 2014). In fact, tDCS applied in this manner worked twice as well and three times as long as caffeine. Additionally, the effects were discovered to be robust across tasks and insensitive to small changes in anode position (McKinley et al., 2015). Other researchers have also discovered that the extracephalic electrode approach produces a large, robust, and replicable effect on cognition (e.g., Clark et al., 2012; Coffman et al., 2012; Falcone et al., 2012). It has been specifically shown to be most useful for enhancements of attention, learning, and memory (Coffman et al., 2014). In fact, it is believed that the effects of tDCS on learning may be driven, at least in part, by enhancements in attention (Clark et al., 2012; McKinley et al., 2013).

Regardless, the extracephalic approach has been shown to have a large, and repeatable effect on attention that may aid in perception of stimuli in multitasking environments.

A limitation with this research is the possible enhancement in other cognitive characteristics, i.e., working memory and how that may have a role in multitasking performance. Recent research has also shown that there is a relationship between working memory and multitasking ability (e.g., Redick, 2016). Rather than performing tasks simultaneously, multitasking involves shifting attention between tasks in a serial fashion (Hambrick et al., 2010; Adler and Benbunan-Fich, 2012). Working memory stores information regarding the tasks attended to most recently allowing the individual to predict which task requires attention next (Taylor et al., 2015a,b). Converging evidence suggests that tDCS applied to the DLPFC improves working memory performance. For example, a study conducted by Andrews evaluated the effects of anodal tDCS administered to the lDLPFC on working memory performance as measured by an n-back task (Andrews et al., 2011). The findings showed that there was a significant improvement in accuracy for the tDCS group compared to the sham group. Similarly, Hoy et al. (2013) found that anodal tDCS applied to lDLFPC generated significant improvements in accuracy during a 2-back test. In fact, in the vast majority of studies examining the effects of tDCS on working memory, the anode is placed over the lDLPFC rather than the right (for a meta-analysis, see Brunoni and Vanderhasselt, 2014). This is largely due to the fact that activation of the left prefrontal cortex was superior compared to the right prefrontal cortex during a cognitive task for right-handed participants (Schambra et al., 2011). Given that many of these tDCS studies used right-handed participants, lDLPFC is the intuitive choice. However, it should be mentioned that stimulation of the rDLPFC may also influence working memory performance. A study conducted by Ruf evaluated the effects of anodal tDCS on working memory via an n-back task when applied to the rDLPFC (Ruf and Plewnia, 2015). Their findings also showed that anodal tDCS had a significant improvement in accuracy compared to the sham group. With the established link between working memory performance and multitasking capability, it follows that the influence of tDCS on working memory may produce performance enhancements in multitasking. For example, tDCS may improve the ability to store information regarding the subtasks most recently executed to appropriately direct attention to the next subtasks that must be accomplished.

Stimulation of the lDLPFC have also been linked to improvements in visual search (Bolognini et al., 2010). Because multitasking involves switching attention between multiple tasks in a serial fashion However, many of the visual search studies involving tDCS have targeted areas of the posterior parietal cortex (e.g., Nitsche et al., 2009). However, we have previously found that tDCS produced visual search improvements via stimulation of the frontal eye fields (FEF) (Nelson et al., 2015). Because of the proximity of the FEF to the prefrontal cortex and the large dispersion of current delivered by tDCS, it was concluded that the effects may have been due to influences in visual attention. In fact, the results matched well with previous investigations into the effects of tDCS on sustained attention using vigilance tasks (e.g., McKinley et al., 2012). Another possibility is that tDCS may have enhanced visual processing efficiency, allowing participants to more rapidly perceive and process visual attention and search more rapidly. For example, tDCS significantly improves response times without a change in response bias on a variety of visually-based cognitive tasks (Fiori et al., 2011; Falcone et al., 2012; McIntire et al., 2014; McKinley et al., 2017). It is likely that this change is a result of improved processing speed. Further, the eye blink data in Nelson et al. (2015) showed elevated blink rates associated with more eye saccades. This supported the hypothesis that tDCS induced a higher attention state and a more active search of the stimuli (i.e., potentially faster processing speed). Improvements in processing speed would inevitably aid multitasking by increasing information throughput.

To date, there have been very few studies that examined the effects of prefrontal tDCS on multitasking directly. Scheldrup et al. (2014) found that anodal tDCS applied to left ventral-lateral prefrontal cortex improved performance on a subtask involving memory for irregularly-appearing symbols. This effect of tDCS was sub-task specific. Modifying the stimulation montage (i.e., placement of the electrodes) changed the subtask that was influenced by stimulation. Hence, it may be possible to only influence portions of the multitask.

Many researchers have provided evidence that transcranial direct current stimulation can be implemented to improve vigilance and sustained attention (Coffman et al., 2012; Nelson et al., 2014; Roe et al., 2016), working memory (Fregni et al., 2005; Brunoni and Vanderhasselt, 2014) and motor coordination skills (Shah et al., 2013) during single task operations. However, there is very little research discussing the effects of transcranial direct current stimulation in a multitasking environment. Thus, the purpose of this study was to determine at what baud rate (difficulty level) a multitasking throughput capacity becomes present and whether transcranial direct current stimulation can improve performance resulting in a higher throughput capacity.

## Materials and methods

### Subject

The study protocol was approved in advance by the Air Force Research Laboratory Institutional Review Board (IRB) for testing on human subjects to evaluate the effects of transcranial direct current stimulation (tDCS) on information processing while performing the multi-attribute task battery (MATB). Limited research has been published on the effects of tDCS to improve human operator multitasking capability, however in a previous study the same performance metrics were evaluated to compare a control vs. feedback condition (Camden et al., 2015). From the analysis of the baud output from this study, the pooled standard deviation of subjects for a pairwise comparison of Condition was 0.11. We felt a mean difference of 0.15 was reasonable with a power equivalent to 0.82 resulting in a per Condition sample size calculation of 10. Extensive experience from previous stimulation research by investigators involved in this study also indicated 10 subjects in a between subject design should be sufficient for the research objectives of this study. Therefore, a total of 20 participants (16 male and 4 female) from Wright-Patterson Air Force Base were randomly assigned to one of the two Conditions (anodal tDCS and sham tDCS) on the testing day with the constraint that each group had gender equality. A between subject design was selected to reduce learning effect from the multi-attribute task battery (MATB). There were eight males and two females in each of the Conditions. The age for the participants ranged from 21 to 41 years old with an average age of 31.1 (SD = 4.5). The age for the anodal tDCS group ranged from 24 to 37 years old with an average age of 30.7 (SD = 3.8) and the age for the sham tDCS group ranged from 21 to 41 years old with an average age of 31.5 (SD = 5.3). Participation in the study was completely voluntary and the participants were able to withdraw at any time if they wished to do so. Participants had to be between the ages of 18–42 years old, speak English and have basic computer skills to be included in the study. The age restriction was enforced to keep the control group homogenous and to reduce possible aging effect. As well, additional screening was conducted to ensure participants did not have any neurological disorders, motor coordination problems, color deficiencies or experience using MATB.

### Equipment

#### Multi-attribute task battery (MATB)

**Figure 2:**
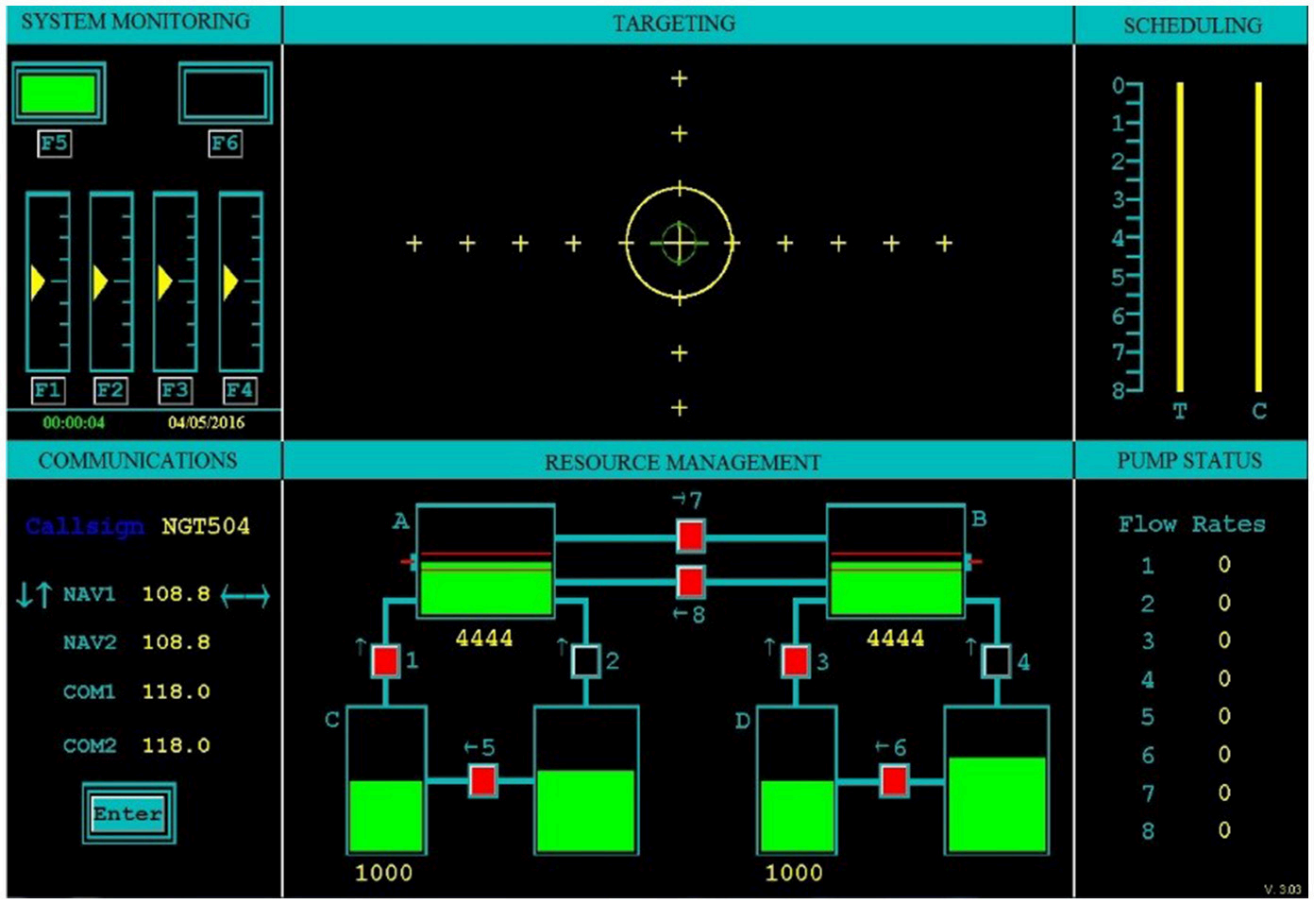
**User interface of the multi-attribute task battery (MATB)**.

The Multi-Attribute Task Battery (MATB) was developed by the National Aeronautics and Space Administration (NASA) to evaluate human performance in a multitasking environment (Comstock and Annegard, 1992). The U.S. Air Force version of MATB (AF-MATB) operating in information throughput (IT) mode was implemented in this study (Miller et al., 2014). AF-MATB requires the human operator to simultaneously monitor and respond to four independent tasks on one computer screen (Figure 2). The task consists of systems monitoring, communication, targeting and resource management. For this study each of the four tasks were equally weighted, therefore no task had greater importance than another task. The following depicts the objectives for each tasks:

System Monitoring: This task was located in the top left corner of the MATB window and consists of two subtasks: lights and dials. The two rectangles at the top represent the lights. The objective of the lights was to keep the left light in the on status “displaying green” and the right light in the off status “displaying black.” If the lights switched from these initial conditions, selecting the F5 or F6 keys reset the lights. Beneath the lights are four vertical columns which consist of dials. Throughout the task, the yellow marker within the dials continuously oscillated one location above and below the center of the dial. Occasionally, the yellow marker shifted toward the top or the bottom of the dial and began oscillating around a new location. When this event occurred, participants were to select the corresponding F1–F4 keys to reset the dials.

Communication: This task was located in the bottom left corner of the MATB window. The objective of the communications was to alter the channel and frequency stated in an audio cueing. An audible message instructed the participants to modify a specific communication channel to a given frequency. The participants navigated to the appropriate channel and set the frequency by selecting the up, down, left and right arrow keys.

Targeting: This task was located in the top right corner of the MATB window. Throughout the task, the green cursor drifted around the window. The objective was to maintain the green cursor within the larger yellow circle by using a joystick.

Resource Management: This task was located in the bottom right corner of the MATB window. The objective of the resource management was to maintain a fluid level within the red-line guidelines. This was accomplished by turning “on” and “off” the reservoir tanks by using the 2 and 4 keys. It is important to note that the fluid level is continuously flowing, therefore the 2 and 4 keys will frequently need to be modified.

Each participant completed the MATB program for a duration of 36 min during their training and testing sessions. During the task, the baud input gradually increased every 4 min resulting in a greater number of events occurring. The total baud input began at 0.6 bits/s and increased to 2.2 bits/s by a factor of 0.2 bits/s every 4 min.

#### Transcranial direct current stimulation (tDCS)

The transcranial direct current stimulation (tDCS) was administered using the MagStim NeuroConn DC Stimulator (MagStim Company Limited; Whitland, UK). This device has the capability to provide a continuous current up to 5000 μA. For safety, there are built-in impedance measurements which turns the device off if the impedance exceeds 50 kΩ. Program codes were entered into the device to allow for a double-blinded study, neither the participant nor researcher was aware if the stimulation code activated the real or sham tDCS until after the study was complete. The active stimulation provided a continuous current of 2 mA for a duration of 30 min. The sham stimulation provided 2 mA of stimulation for only 30 s. The sham condition emulates the subjective skin sensations present in active tDCS. Both active and sham stimulation ramped the current up and down slowly (i.e., 15 s ramps). A study conducted by Boggio evaluated current density levels during a working memory task (1 mA vs. 2 mA) and found that 2 mA tDCS provided significant enhancement compared to 1 mA (Boggio et al., 2006). For this reason, a current density of 2 mA was selected for the study.

The electrodes used in this study were pads consisting of 5 silver/silver chloride electroencephalographic (EEG) electrodes arranged in a circular pattern (Rio Grande Neurosciences, Santa Fe, NM, USA) compared to the traditional 5 × 7 cm wet sponge electrodes. These electrodes are described in detail in a previous study (McKinley et al., 2013). It has been shown that the EEG electrodes have greater stability, lower sensations and less skin irritation compared to the wet sponge electrodes (Nelson et al., 2015). When providing 2 mA of continuous current, the average current density was 0.199 mA/cm^2^. Several studies have now shown that applying tDCS with the EEG electrodes can improve various cognitive functions such as visual search detection accuracy (Nelson et al., 2015), accelerated learning (McKinley et al., 2013), procedural learning (McKinley et al., 2014), and working memory and vigilance (McIntire et al., 2014).

A between subjects experimental design was utilized with one factor (tDCS type) tested at two levels: anodal tDCS and sham tDCS. The reason for selecting a between subject experimental design was to reduce learning effect from the multi-attribute task battery (MATB). The anodal tDCS group received 2 mA of tDCS for a duration of 30 min over the left dorsolateral prefrontal cortex during the testing session. The sham tDCS group received tDCS for 30 s over the left dorsolateral prefrontal cortex during the testing session. The left dorsolateral prefrontal cortex was selected as the stimulation site because this region of the brain is associated with sustained attention, working memory, decision making, planning and reasoning which are all directly involved with multitasking (Pochon et al., 2001; Javadi and Walsh, 2012).

The Human Operator Informatic Model (HOIM) was one of the first mathematical models developed that evaluates both human performance and strategy during a multitasking environment (Phillips et al., 2007, 2013). The detailed mathematical equations have recently been summarized for AF-MATB (Camden et al., 2015). The model defines the amount of information input displayed during a multitasking environment as the baud input rate (β_*IN*_). When the human operator correctly responds to the information input during the task, a baud output (β_*O*_) is recorded. Given these variables, the information throughput (β) or the human operators processing capability can be calculated by the ratio of either the total baud output divided by the total baud input (overall throughput) or each task-specific baud output divided by its respective baud input (task-specific throughput). Following this mathematical model, we will be able to determine the amount of information an operator can process before the throughput capacity limits are reached.

### Procedures

The study took place over two consecutive days. On the first visit, each of the subjects were briefed on the informed consent document (ICD) which depicts the nature and purpose, procedures and risks of the study. It is important to note that no research activities commenced until the research subject's questions were answered and the informed consent document was signed. A background and medical screening questionnaire were completed to ensure the subjects qualified to participate in the study. Following the completion of the forms, each subject was provided with a powerpoint slide that provided a description and instructions for each individual task. The first visit was considered training and each of the subjects completed the nine 4 min segments of the multi-attribute task battery (MATB) without the transcranial direct current stimulation to become familiar with the MATB program and difficulty level. The first segment used a baud input (β_*IN*_) rate of 0.6 bits/s. This baud rate increased by a factor of 0.2 bits/s for each of the following segments. The objective of training is to reach a performance asymptote for the MATB program for each subject. If a performance asymptote is not observed, the subject will not continue in the study. Once the 36 min task was completed, each subject was able to leave for the day.

The second visit was considered the testing day where the subjects were either provided with anodal or sham tDCS while performing the MATB program depending on their assigned condition.

### Statistical analysis

Statistical analysis was conducted using the statistical analysis system (SAS version 9.2). For the overall and each individual task components from the MATB sessions (system monitoring, communication, targeting, and resource management) an analysis of variance was performed using baud output and throughput capacity as dependent variables with tDCS condition (anodal and sham) as a between factor and baud input (9 levels) as a within factor. Pairwise comparisons of condition at each baud input were performed using two-tailed two-sample t-tests. It is important to note that the throughput capacity is the percent efficiency in relation to the baud output and baud input. Therefore, the *t*-test comparisons for the anodal and sham tDCS groups at each baud input rate will be identical for baud output and throughput capacity. No error level adjustment was made for the number of tests but *p*-values are provided. Cohen's d effect size was also determined.

## Results

The results for the study were analyzed focusing on two different sections: overall output and throughput and the individual tasks output and throughput for each of the nine baud input rates. We will begin by discussing the overall output and information throughput followed by the individual task components output and information throughput for the MATB program.

**Figure 3:**
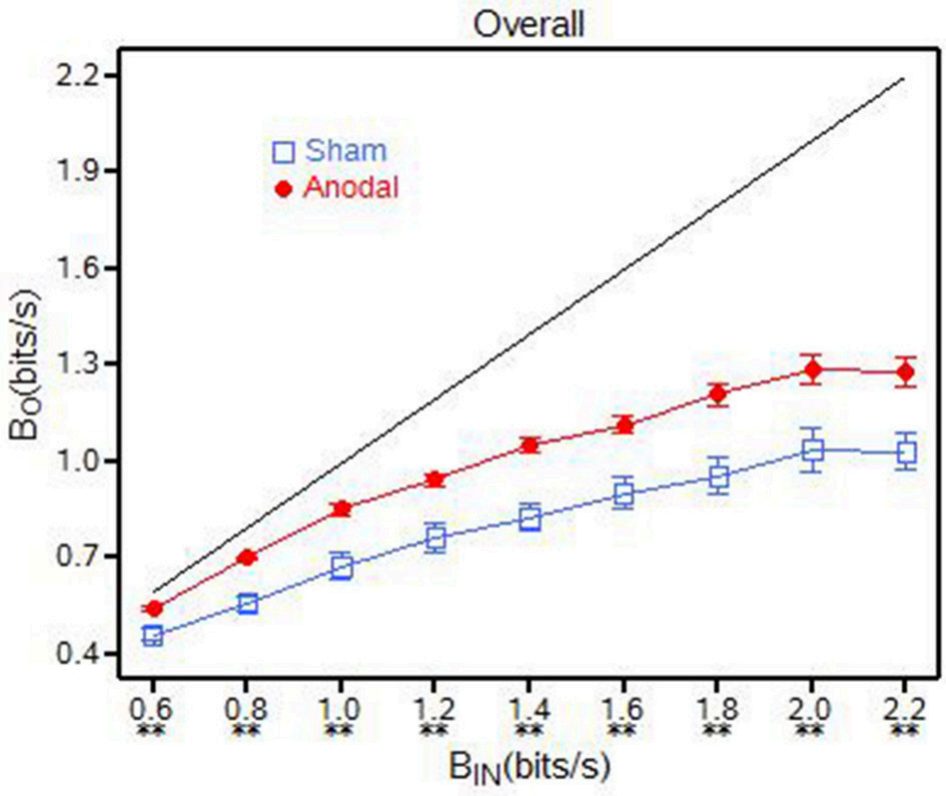
Overall baud (β_***O***_) for the anodal and sham tDCS groups, ^**^p < 0.01; ^*^ 0.01 < *p* < 0.05.

As shown in Figure 3, there was a significant interaction [*F*_(8, 144)_ = 3.10, *p* = 0.0030] in overall baud output (β_*O*_) between the anodal and sham tDCS groups and the baud input rates (β_*IN*_). Analysis of Variance (ANOVA) results for the overall task are shown in Table 1. The means for the anodal tDCS group were statistically higher (*p* ≤ 0.0069) than those of the sham tDCS group at each of the baud input rates. It appears that the baud output (β_*O*_) was approaching a multitasking throughput capacity near 1.3 bits/s (for the anodal tDCS group) and near 1.0 bits/s (for the sham tDCS group) when β_*IN*_ approached 2.0 bits/s. The comparisons at each of the nine baud input rates are listed in Table 2.

**Table 1:** **Analysis of Variance depicting the overall performance for the multi-attribute task battery (MATB)**.

**Performance metric**	**Source**	**β_*O*_ (bits/s)**		**Throughput capacity (%)**	
		***F***	***p***	***F***	***p***
Total	Condition	17.94	0.0005	20.13	0.0003
	Input	201.84	0.0001	118.99	0.0001
	Condition^*^Input	3.10	0.0030	1.41	0.1967

All F-tests of Condition had DF = 1.18 and all F-tests of Input and Condition^*^Input had DF = 8.144.

**Table 2:** **Comparison of groups at each baud rate**.

**β_*IN*_(bita/s)**	**Anodal**				**Sham**				**Two-tailed Two-sample *t*-test**			**Cohen's d**
	**β_*O*_ (bits/s)**		**Throughput (%)**		**β_*O*_ (bits/s)**		**Throughput (%)**					
	**Mean**	**SEM**	**Mean**	**SEM**	**Mean**	**SEM**	**Mean**	**SEM**	**DF**	***t***	***p***	
0.6	0.543	0.005	89.3	0.8	0.462	0.018	75.9	2.9	10.2	4.43	0.0012	2.09
0.8	0.700	0.007	87.0	0.9	0.558	0.023	69.4	2.9	10.6	5.86	0.0001	2.76
1.0	0.853	0.019	83.3	1.8	0.675	0.039	65.9	3.9	12.9	4.08	0.0013	1.92
1.2	0.943	0.020	77.0	1.7	0.761	0.047	62.1	3.8	12.3	3.56	0.0038	1.68
1.4	1.053	0.024	74.2	1.7	0.827	0.044	58.3	3.1	18.0	4.53	0.0003	2.13
1.6	1.115	0.025	69.0	1.6	0.902	0.049	55.8	3.0	18.0	3.87	0.0011	1.83
1.8	1.208	0.036	66.4	2.0	0.956	0.059	52.5	3.3	18.0	3.63	0.0019	1.71
2.0	1.287	0.042	64.1	2.1	1.037	0.070	51.6	3.5	18.0	3.05	0.0069	1.44
2.2	1.279	0.047	58.1	2.1	1.031	0.057	46.8	2.6	18.0	3.34	0.0037	1.57

**Figure 4:**
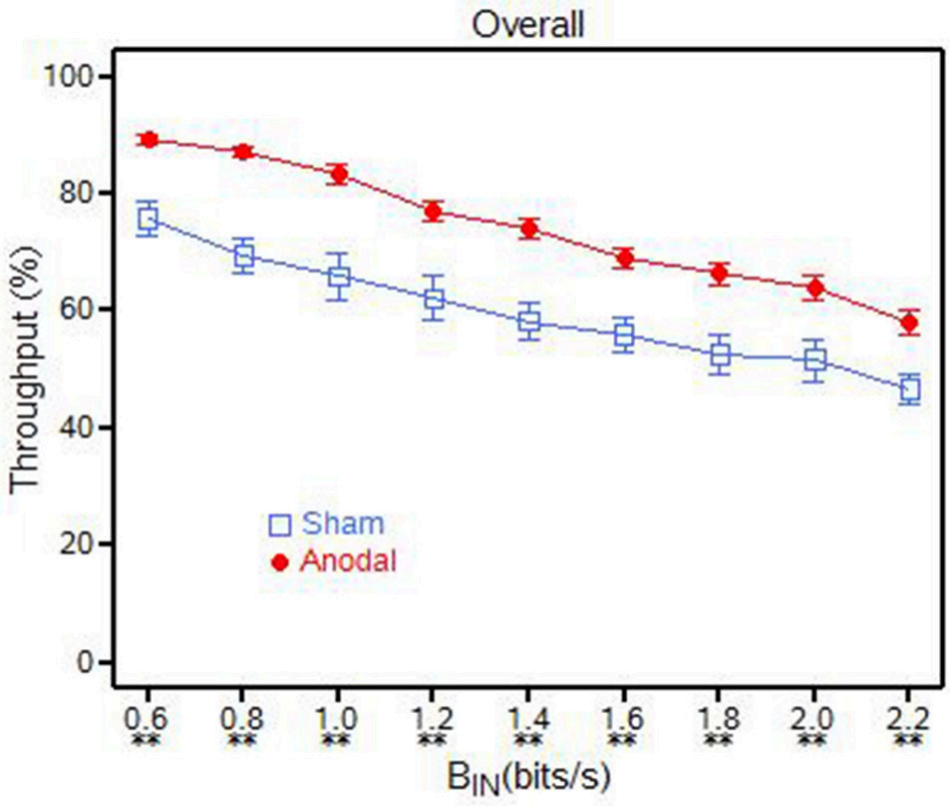
Overall throughput (β) for the anodal and sham tDCS groups, ^**^p < 0.01; ^*^0.01 < ***p*** < 0.05.

The information throughput (β) percentage was significantly higher [*F*_(1, 8)_ = 20.13, *p* = 0.0003] for the anodal tDCS group compared to the sham tDCS group across all baud input rates (Table 1 and Figure 4). The β for the anodal tDCS group was between 11 and 17% higher than the sham tDCS group. The means and *p*-values for these *t*-tests are presented in Table 2.

The ANOVA results for each individual component are shown in Table 3 with the means, standard deviations and *p*-values for each comparison of the individual components shown in Table 4.

**Table 3:** **Analysis of Variance depicting the individual tasks performance for the multi-attribute task battery (MATB)**.

**Performance metric**	**Source**	**β_*O*_ (bits/s)**		**Throughput capacity (%)**	
		***F***	***p***	***F***	***p***
System Monitoring	Condition	15.02	0.0011	15.67	0.0009
	Input	70.52	0.0001	4.92	0.0001
	Condition^*^Input	3.36	0.0015	0.52	0.8395
Communication	Condition	2.74	0.1152	2.80	0.1116
	Input	35.42	0.0001	9.34	0.0001
	Condition^*^Input	1.16	0.3301	0.89	0.5283
Targeting	Condition	1.44	0.2455	2.15	0.1595
	Input	18.94	0.0001	139.87	0.0001
	Condition^*^Input	0.21	0.9879	0.70	0.6925
Resource Management	Condition	8.03	0.0110	11.06	0.0038
	Input	202.86	0.0001	84.09	0.0001
	Condition^*^Input	2.11	0.0381	11.56	0.0001

All F-tests of Condition had DF = 1.18 and all F-tests of Input and Condition^*^Input had DF = 8.144.

**Table 4:** **Comparison of individual tasks at each baud rate**.

**Dependent variable**	**β_IN_ (bits/s)**	**Anodal**				**Sham**				**Two-tailed Two-sample *t*-test**			**Cohen's d**
		**β_*O*_ (bits/s)**		**Throughput (%)**		**β_*O*_ (bits/s)**		**Throughput (%)**					
		**Mean**	**SEM**	**Mean**	**SEM**	**Mean**	**SEM**	**Mean**	**SEM**	**DF**	***t***	***p***	
System Monitoring	0.6	0.127	0.006	84.7	4.2	0.094	0.012	62.8	7.8	18.0	2.49	0.0230	1.17
	0.8	0.163	0.009	81.5	4.4	0.109	0.013	54.4	6.5	18.0	3.47	0.0027	1.64
	1.0	0.209	0.010	83.7	3.9	0.127	0.017	50.8	6.9	18.0	4.11	0.0007	1.94
	1.2	0.229	0.013	76.4	4.5	0.146	0.018	48.7	6.0	18.0	3.70	0.0017	1.74
	1.4	0.258	0.017	73.8	4.7	0.175	0.017	50.0	4.8	18.0	3.53	0.0024	1.67
	1.6	0.294	0.013	73.4	3.2	0.195	0.021	48.9	5.3	18.0	3.98	0.0009	1.87
	1.8	0.327	0.017	72.7	3.7	0.213	0.028	47.3	6.2	18.0	3.51	0.0025	1.66
	2.0	0.374	0.022	74.8	4.4	0.250	0.036	49.9	7.2	18.0	2.93	0.0090	1.38
	2.2	0.390	0.021	70.9	3.9	0.255	0.040	46.4	7.2	18.0	3.01	0.0076	1.42
Communication	0.6	0.138	0.003	96.7	2.2	0.131	0.010	91.7	6.7	11.0	0.71	0.4929	0.33
	0.8	0.188	0.002	98.8	1.2	0.166	0.011	87.5	5.9	9.8	1.87	0.0919	0.88
	1.0	0.249	0.008	95.5	3.1	0.207	0.024	79.1	9.3	11.0	1.67	0.1232	0.79
	1.2	0.280	0.009	90.8	3.0	0.247	0.029	80.0	9.5	10.8	1.08	0.3018	0.51
	1.4	0.333	0.008	93.3	2.2	0.257	0.029	72.0	8.0	10.4	2.57	0.0272	1.21
	1.6	0.359	0.011	88.8	2.8	0.299	0.041	74.1	10.1	10.4	1.40	0.1908	0.66
	1.8	0.401	0.017	88.9	3.8	0.306	0.050	67.9	11.2	11.1	1.79	0.1016	0.84
	2.0	0.425	0.019	85.2	3.9	0.333	0.065	66.7	12.9	10.6	1.38	0.1972	0.65
	2.2	0.399	0.032	73.0	5.9	0.328	0.063	60.0	11.5	18.0	1.01	0.3250	0.48
Targeting	0.6	0.156	0.003	94.2	1.6	0.146	0.004	88.1	2.1	18.0	2.32	0.0324	1.09
	0.8	0.198	0.004	92.6	1.7	0.178	0.007	83.2	3.2	18.0	2.62	0.0172	1.24
	1.0	0.225	0.009	85.6	3.3	0.202	0.011	77.0	4.2	18.0	1.62	0.1227	0.76
	1.2	0.242	0.008	76.4	2.7	0.214	0.012	67.8	3.7	18.0	1.90	0.0736	0.90
	1.4	0.258	0.009	71.4	2.6	0.223	0.016	61.9	4.5	18.0	1.85	0.0808	0.87
	1.6	0.248	0.014	60.3	3.4	0.224	0.021	54.3	5.1	18.0	0.98	0.3386	0.46
	1.8	0.255	0.017	54.6	3.7	0.236	0.022	50.4	4.7	18.0	0.71	0.4888	0.33
	2.0	0.256	0.018	50.4	3.6	0.239	0.029	46.9	5.8	18.0	0.50	0.6222	0.24
	2.2	0.242	0.018	43.5	3.2	0.222	0.031	40.0	5.5	18.0	0.55	0.5862	0.26
Resource Management	0.6	0.122	0.005	81.5	3.0	0.091	0.006	60.8	4.1	18.0	4.07	0.0007	1.92
	0.8	0.151	0.006	75.4	3.1	0.105	0.009	52.5	4.4	18.0	4.26	0.0005	2.01
	1.0	0.170	0.006	67.9	2.5	0.139	0.007	55.6	2.8	18.0	3.26	0.0044	1.54
	1.2	0.191	0.006	63.9	2.0	0.154	0.009	51.2	2.9	18.0	3.58	0.0021	1.69
	1.4	0.204	0.009	58.1	2.5	0.172	0.010	48.9	3.0	18.0	2.34	0.0307	1.11
	1.6	0.214	0.009	53.5	2.2	0.184	0.008	46.0	2.1	18.0	2.49	0.0227	1.17
	1.8	0.224	0.010	49.8	2.3	0.201	0.009	44.6	2.1	18.0	1.65	0.1172	0.78
	2.0	0.231	0.007	46.3	1.4	0.216	0.014	43.1	2.7	18.0	1.02	0.3210	0.48
	2.2	0.248	0.008	45.1	1.4	0.226	0.010	41.1	1.9	18.0	1.73	0.1016	0.81

**Figure 5:**
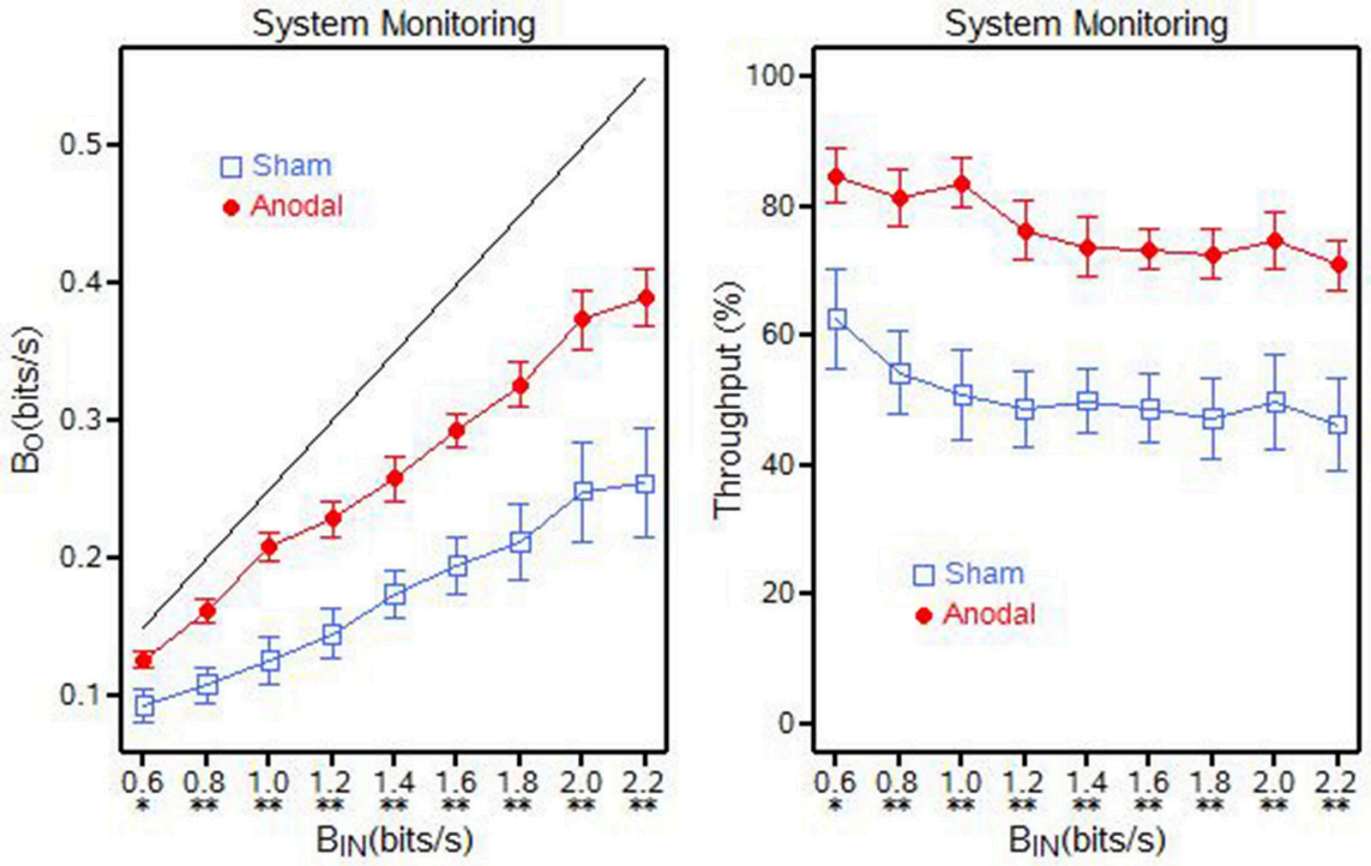
System monitoring output (β_***O***_) and throughput (β) for the anodal and sham tDCS groups, ^**^p < 0.01; ^*^0.01 < *p* < 0.05.

For System Monitoring, it appears that the baud output (β_*O*_) was approaching a throughput capacity near 0.4 bits/s (for the anodal tDCS group) and near 0.26 bits/s (for the sham tDCS group) when baud input (β_*IN*_) approached 2.0 bits/s. The results provide evidence that anodal tDCS significantly increases the baud output (β_*O*_) and information throughput (β) (See Figure 5). Each of the nine baud input rates displayed a significant difference in groups (*p* = 0.0230).

**Figure 6:**
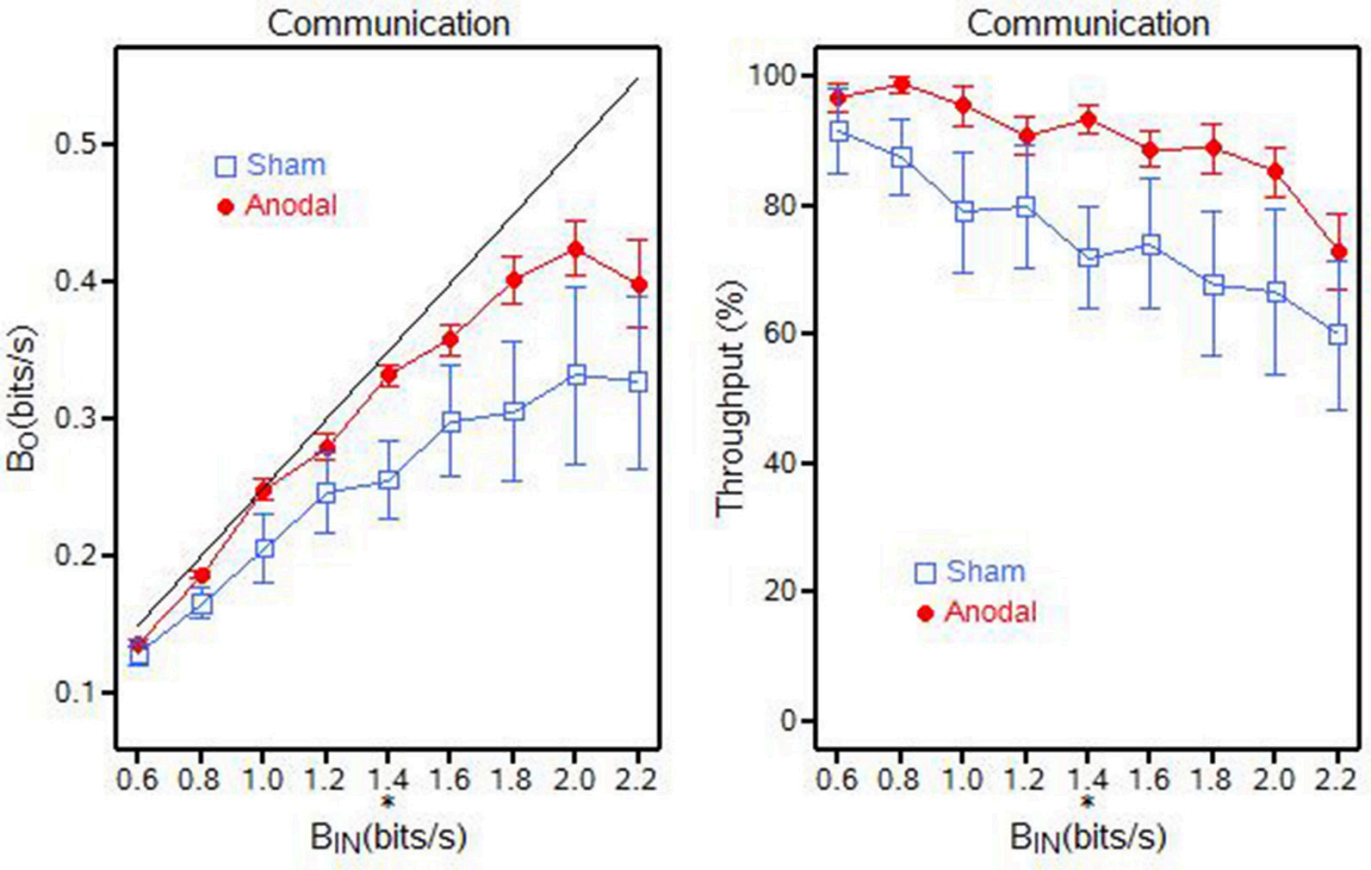
Communication output (β_***O***_) and throughput (β) for the anodal and sham tDCS groups, ^**^p < 0.01; ^*^0.01 < *p* < 0.05.

For Communications, it appears that the baud output (β_*O*_) was approaching a throughput capacity near 0.43 bits/s (for the anodal tDCS group) and near 0.33 bits/s (for the sham tDCS group) when baud input (β_*IN*_) approached 2.0 bits/s. The results suggest that baud output (β_*O*_) and information throughput (β) were statistically greater (*p* = 0.0272) for the anodal tDCS group when compared to the sham tDCS group, when the baud input rate was 1.4 bits/s (Figure 6). There were no significant differences at any of the other baud input rates.

**Figure 7:**
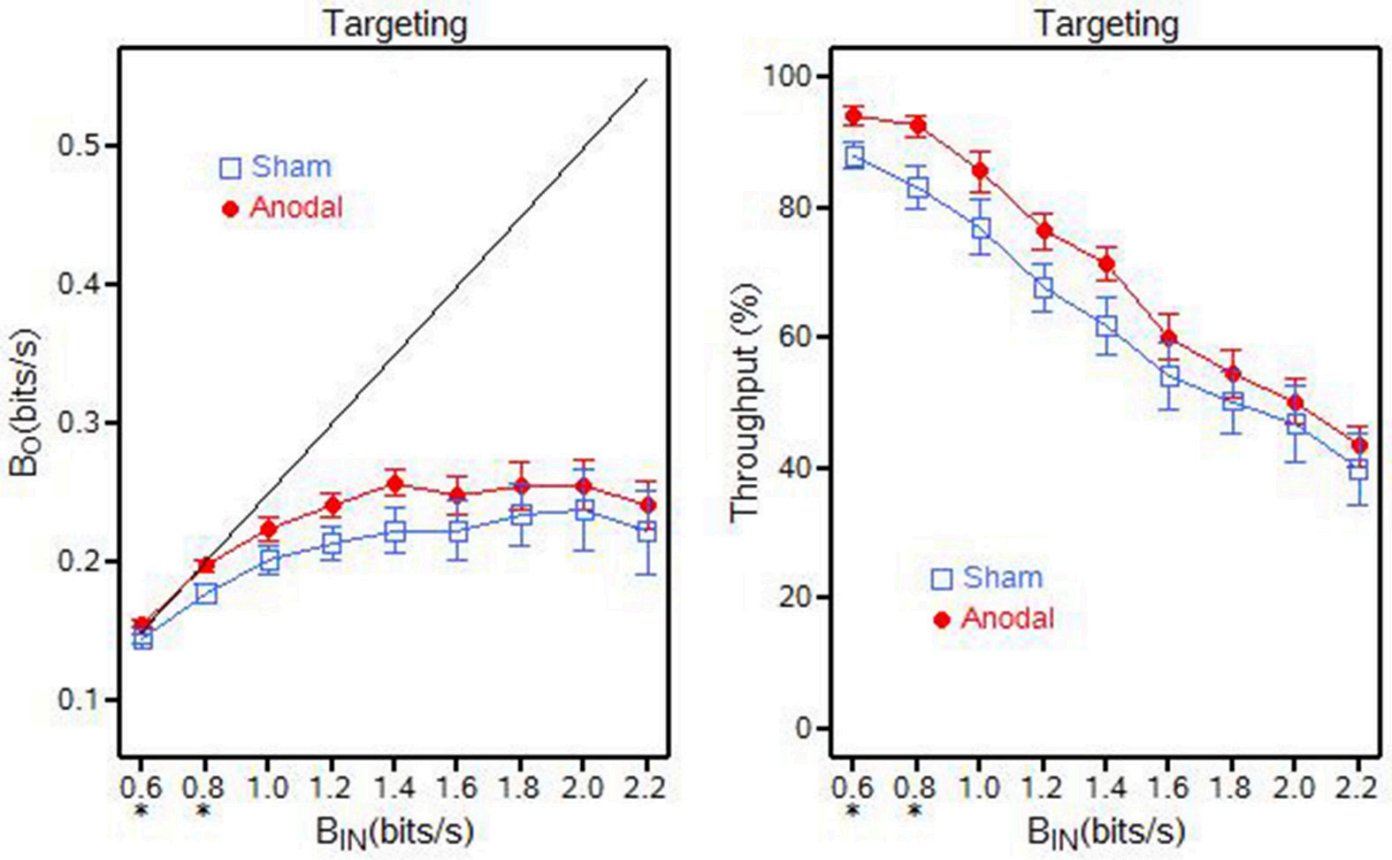
Targeting output (β_***O***_) and throughput (β) for the anodal and sham tDCS groups, ^**^p < 0.01; ^*^0.01 < *p* < 0.05.

For Targeting, it appears that the baud output (β_*O*_) was approaching a throughput capacity near 0.26 bits/s when baud input (β_*IN*_) approached 1.4 bits/s (for the anodal tDCS group) and near 0.24 bits/s when baud input (β_*IN*_) approached 2.0 bits/s (for the sham tDCS group). The baud output (β_*O*_) and information throughput (β) were statistically greater for the anodal tDCS group at the baud rate of 0.6 and 0.8 bits/s (*p* ≤ 0.0324) when compared to the sham tDCS group (Figure 7). There were no significant differences at any of the other baud input rates.

**Figure 8:**
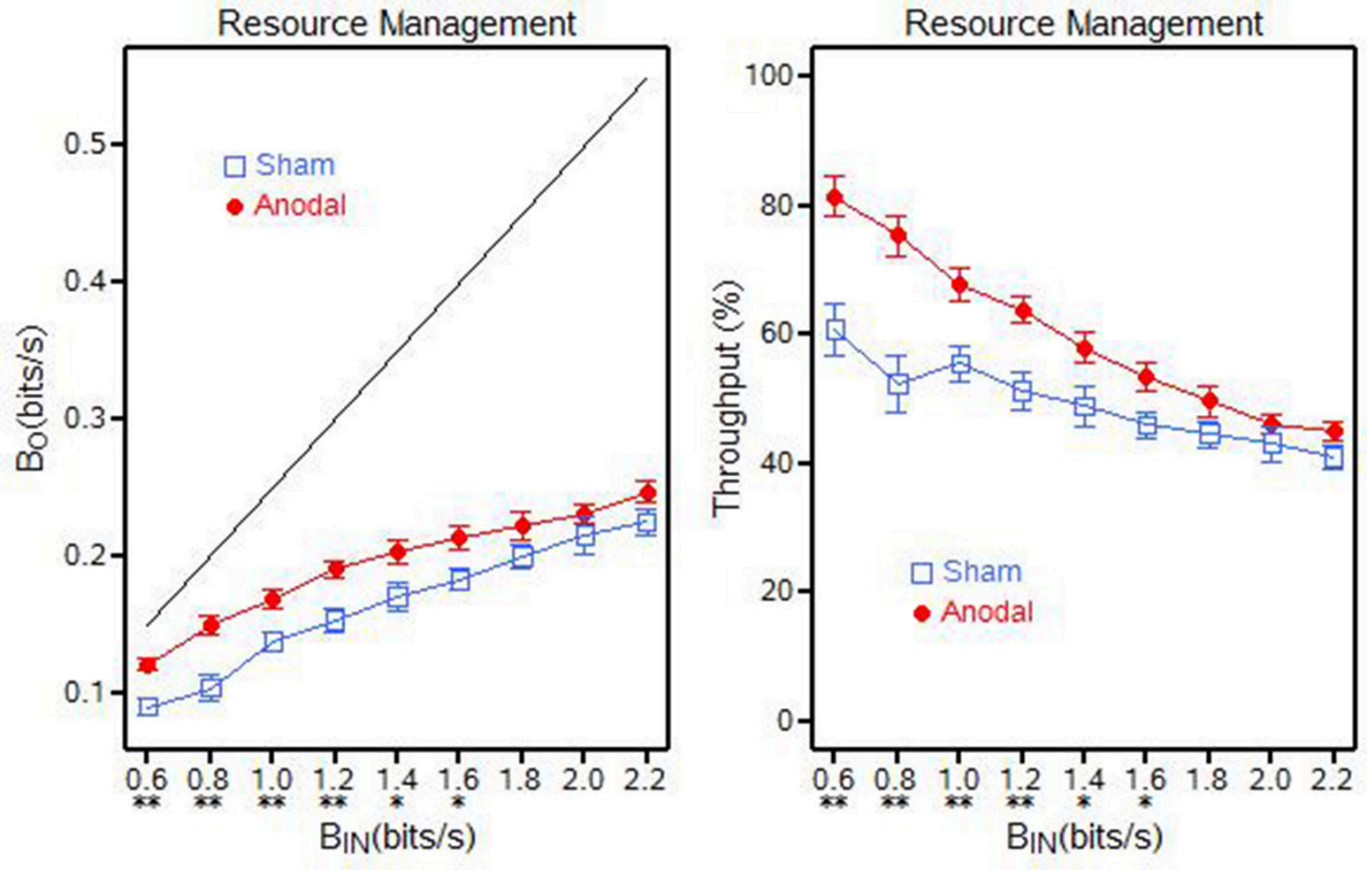
Resource management output (β_***O***_) and throughput (β) for the anodal and sham tDCS groups, ^**^p < 0.01; ^*^0.01 < *p* < 0.05.

Resource Management did not display a throughput capacity. The findings show that at the lower level baud rates (0.6, 0.8, 1.0, 1.2, 1.4, and 1.6 bits/s) there was a statistically significance difference between the baud outputs for the anodal and sham tDCS groups at each of the baud input levels (*p* ≤ 0.0307). The difference in baud outputs for the highest three baud input rates were not significant (Figure 8).

## Discussion

The ability for a human operator to multitask efficiently in a military setting has been a major issue for the past several decades. Lower sensor and display costs have resulted in a large increase in the information that can be presented to the human operator. Consistent with Miller for single-tasking, we found that when information throughput becomes complex and overwhelming a multitasking throughput asymptote will occur (Miller, 1956). Many efforts have been made to improve information processing efficiency and/or vigilance to surpass the throughput capacity limitation such as caffeine and pharmaceutical medication. However, these countermeasures are short lived and display side effects that may negatively influence cognitive performance, alertness and mood.

Transcranial direct current stimulation (tDCS) has readily become a key component in augmenting and improving cognition in single and multitask scenarios. In a recent study, applying anodal tDCS to the left dorsolateral prefrontal cortex improved multitasking in a 3D video game simulation compared to a sham tDCS group (Hsu et al., 2015). Although the findings showed cognitive enhancement occurred in the latter stages of the task, there may be a delayed effect when receiving tDCS on the left dorsolateral prefrontal cortex while multitasking. On the contrary, a study conducted by Filmer found that applying cathodal tDCS to the left posterior lateral prefrontal cortex (lpLPFC) improved multitasking capabilities with respect to reaction time compared to anodal and sham tDCS (Filmer et al., 2013). Our data suggest a more immediate effect (i.e., within 4 min) when applying 2 mA of anodal tDCS to the lDLPFC during the MATB task. Similar to the findings of Scheldrup et al. (2014), the effects were larger for specific subtasks. In particular, the subtasks that tested sustained attention and vigilance (i.e., system monitoring and resource management) displayed a greater enhancement compared to the auditory and motor coordination tasks (i.e., communications and targeting). This is not surprising given that the applied tDCS montage has been repeatedly shown to positively influence performance in a variety of sustained attention/vigilance tasks (McIntire et al., 2014, 2017; Nelson et al., 2015). The data from the experiment reported herein provide new evidence that attention/vigilance performance is enhanced even when attention is divided among multiple tasks. This potentially has important implications for high workload environments that provide information to the operator via a wide range of stimuli. Additional research should be conducted to evaluate the robustness of these observed effects.

In addition to improvements in sustained attention, the applied tDCS montage has been previously shown to induce increased arousal and wakefulness during 30 or 36 h of sleep deprivation (McIntire et al., 2014, 2017). Given that the LC regulates attention, arousal, and wakefulness, it is possible that the tDCS paradigm is causing changes in subcortical brain regions including the LC. Recently, Boasso et al. (2016) provided evidence that transdermal stimulation of the trigeminal nerve influences LC activity. Importantly, the trigeminal nerve has projections into the forehead very close to the anode placement used in our lDLPFC stimulation montage. Perhaps more interesting is that both Boasso et al. (2016) and McIntire et al. (2014) produced similar improvements in mood as measured by the profile of mood states (POMS). Hence, it is possible that the tDCS paradigm used in McIntire et al. (2014) and Nelson et al. (2015) is simply stimulating projections of the trigeminal nerve and causing alterations in LC activity downstream. Another possibility is that deeper structures such as the LC may have been modulated by the unique tDCS electrode montage. There is evidence that increasing the distance between electrodes can increase delivered current and brain modulation due to less scalp shunting (Moliadze et al., 2010). By having an extracephalic cathode, the current may be forced along a deeper pathway through the brain, exciting subcortical regions along the way. However, additional experiments that include neuroimaging would be needed to test this hypothesis. Either way, the behavioral data suggests possible LC involvement that should be a focus of future studies.

With an established relationship between working memory and multitasking ability (e.g., Redick, 2016) coupled with evidence that tDCS enhances working memory (e.g., Andrews et al., 2011), an enhancement of multitasking ability through working memory enhancement was expected. When searching the computer screen for new critical stimuli, working memory serves to store the areas of the screen that have already been observed to allow the participant to more efficiently search the remaining scene (Biggs et al., 2013). In fact, Biggs et al. (2013) showed that consistent searchers perform better at accurately detecting stimuli than inconsistent searchers purportedly by alleviating the memory burden associated with visual search. Hence, improvements in working memory should have an equal effect across the subtasks by aiding the operator in determining what portion of the screen to attend to next. While performance in the communication and targeting tasks was enhanced for the anodal tDCS group compared to the sham tDCS, the level of effect was very weak compared to that observed in the system monitoring and resource management task. Thus, tDCS did not impact all four tasks equally. This indicates that if working memory improvements (if present) had minimal influence on overall behavior. Instead, the applied tDCS paradigm may preferentially improve performance in vigilant based multitasking subtasks, indicating a larger role of tDCS-induced attention benefits.

Recently, Brem et al. (2014) postulated that tDCS-induced increases in brain activation may lead to an increase in processing power to the affected regions. Further, there is a substantial evidence that behavioral effects persist long after the stimulation has ceased, likely caused by changes in plasticity (Nitsche and Paulus, 2001; Ferrucci et al., 2009; Brunoni et al., 2010). Single sessions of tDCS have been shown to induce long-term potentiation (LTP) and the LTP-like effects on plasticity within neural networks engaged in executing the cognitive task have been proposed as the underlying mechanism causing enduring after-effects (Rohan et al., 2015). Hence, excitability changes in lDLPFC during tDCS that potentially increase processing power likely result in lasting plastic changes in the engaged frontal networks that create stronger and more efficient synaptic connections. This would, in turn, improve processing efficiency within this network. Behavioral data in the literature seem to support this hypothesis. Specifically, tDCS has been shown to significantly reduce response times a variety of visually-based cognitive tasks without increases in errors (Fiori et al., 2011; Falcone et al., 2012; McIntire et al., 2014; McKinley et al., 2017). While increased arousal could explain this phenomenon, it is possible that increased processing efficiency is also a contributor. The data from the current experiment suggest that a channel capacity exists around a stimulus input rate of 2.0 bits/sec. However, application of anodal tDCS over lDLPFC increased throughput capacity by 11–17% over the sham tDCS group. Part of this increased capacity may be due to increased processing power or efficiency in the attention networks.

With the world becoming more evolved and complex, multitasking is becoming extremely prevalent in everyday society. The findings provided by this and previous studies display evidence that tDCS can enhance sustained attention performance. Further, this study provides suggests these effects are present even when performing multiple tasks simultaneously. The observed behavioral improvements may have been caused by a combination of modulation of the LC, increased processing power/efficiency in the attentional networks, and changes in working memory capacity. Regardless, the evidence suggest that the tDCS montage used has a profound effect on attention-based subtasks. Future research should be focus on examining the role of LC, the influence of pre-stimulation working memory performance on multitasking outcome measures, and the longevity of the effects of tDCS. Additional studies should also examine the robustness and reproducibility of these effects as these results are exploratory and would need to be applied to larger sample sizes.

## Ethics statement

Air Force Research Laboratory Institutional Review Board. Before any specific procedures were performed, potential participants received an informed consent briefing along with a written copy of the consent document to review. Potential participants who did not demonstrate the ability to understand or the willingness to sign the written informed consent document were excluded from the study.

## Author contributions

JN, RM, CP, LMc, CG, AK, and LMo were directly associated with developing the design and methodology of the research study, conducting participant training, and testing, collecting and analyzing the data, assisting with writing and revisions of the manuscript, granted final approval of the content and agreeing to be accountable for all aspects of the work in ensuring questions related to the accuracy and integrity of any part of the work are appropriately investigated and resolved.

## Funding

Funding for the research study was supported by the Air Force Research Laboratory (AFRL) and the Air Force Office of Scientific Research (AFOSR) grant number 17RHCOR485_v1.

### Conflict of interest statement

The authors declare that the research was conducted in the absence of any commercial or financial relationships that could be construed as a potential conflict of interest.
